# A systematic review on reminder systems in physical therapy 

**DOI:** 10.22088/cjim.9.1.7

**Published:** 2018

**Authors:** Majid Jangi, Cesar Ferandez-de-las-Penas, Mahmoud Tara, Fateme Moghbeli, Fariba Ghaderi, Khodabakhsh Javanshir

**Affiliations:** 1 Department of Medical Informatics, School of Medicine, Mashhad University of Medical Sciences, Mashhad, Iran; 2 Department of Physical Therapy, Occupational Therapy, Rehabilitation and Physical Medicine, Universidad Rey Juan Carlos, Alcorcón, Madrid, Spain; 3 Department of Health Information Management, School of Health Management and Information Sciences, Iran University of Medical Sciences, Tehran, Iran; 4 Department, Faculty of Rehabilitation, Tabriz University of Medical Sciences, Tabriz, Iran; 5 Mobility Impairment Research Center, Health Research Institate, Babol University of Medical Sciences, Babol Iran

**Keywords:** Reminder, Physical therapy, Systematic review

## Abstract

**Background:**

The main goal of physical therapy is to help the patient gain a better health status. Several studies have investigated the use of reminders to prevent such failures on the patients’ side. This article presents a systematic review of the literature concerning reminders in physical therapy.

**Methods:**

Databases were searched until May 2017 and literatures were found from April 1992 until 2017. The literature recruitment strategy was based on applying several keywords and Medical Subject Heading (MeSH) combination running against title and abstract, including concepts such as reminder, physical therapy. The finally selected articles were categorized through reminder aspects such as how, who feedback. Data were extracted according to PRISMA guidelines.

**Results:**

In 47% of studies, the reminder was sent to the patients, 29% to the physical therapists and 12% to the caretaker team. In 24% of the studies, paper-based letters were main medium for reminders while the rest were various types of media like emails and SMS mobile text messages. 35% of the articles showed positive effects of the reminders.

**Conclusions:**

Many reminder methods consisted of SMS, phone calls, letters, emails and notices on the wall were used in physical therapy. Reminders may be used to improve patients' adherence to exercise programs.

The main goal of physical therapy is to help the patient to gain a better health status and normal living. The programs include patient’s regular visits applying various therapeutic procedures including instrument-based therapies using ultrasound, transcutaneous electrical nerve stimulation, and mechanical traction (1-5). As a complementary care plan, in most cases, patients are asked to do regular exercises at home and do activities of daily living (ADL) in proper/correct and safe positions (6). For better care management, the patients are also asked to report specific clinical signs and symptoms such as pain, to the therapist (7). It is clinically observed that some patients frequently forget the visiting sessions or the exercise plan. Several studies have investigated the use of reminders to prevent such failures on the patients’ side (8). On the other side, reminders could also be sent to physiotherapists to encourage them to follow the guidelines and care protocols (9). Variety of reminder methods has been researched across literature including short message system, stationary telephone, or emails (8-10). It is expected that proper follow-ups using patient’s reminders in promoting the right and timely exercise could improve the overall outcome. Such reminders could also be sent to the physical therapist as a decision advisor to help them follow the appropriate guidelines. The aim of this paper was to review the scientific literature on the use of reminders in physical therapy to address both sides of the patient and therapist. 

## Methods

**Data sources: **A literature search of studies that investigated the use of patient or therapist reminders in physical therapy (or both) was conducted until May 2017 using Pub Med and Science Direct database found from April 1992 to Mar 2017. The search terms included MeSH (Medical Subject Headings) keywords “physical therapy” or “physiotherapy” or “exercise physical activity” or “low back pain” or “shoulder pain” or “neck pain” or and “knee pain” and reminder and reminders or remind. The literature search was conducted following the guidelines described by Greenhalgh (11). Furthermore, the reference list of the finally selected articles was checked out to find additional relevant studies. The reviewers made every effort to include all relevant articles. 

**Figure1:**
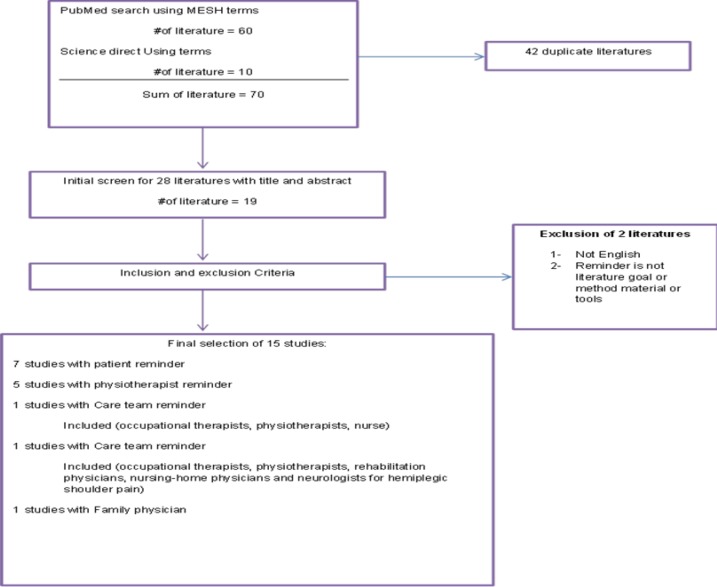
Flow chart of the study selection process

**Article selection: **The articles were evaluated by three reviewers independently. Any disagreements on the eligibility of the articles were resolved getting help from a third research. In the first step, the articles were selected if the word "reminder" was included in the title or abstract by scripts. Then, the word "reminder" was matched separately with other keywords mentioned above (physical therapy, exercise physical activity, low back pain, shoulder pain, neck pain or knee pain) and a more profound review of articles was conducted to select articles which matched to the present title. The researchers selected the articles that: 1- had reminder as their topic of focus. 2- used or researched reminders to remind patients: to perform exercises, to report pain or other symptoms or to remember the next physical therapy appointment; and 3- used or researched reminders to remind physical therapists on how to manage their patients or improve their therapeutic decisions. Articles in languages other than English were not included (figure 1). 

**Data extraction: **A standardized form with questions about sample size, method of assessment, outcome measures and results was used for data extraction according to STARLITE guidelines (12). The parameters of interest were: how to remind? , who and what was reminded? , and what was the feedback of the reminder? We analyzed and categorized the selected articles (how, who, feedback) and compared the outcomes using Microsoft Excel 2015.

## Results

**Article search: **From the forty-one articles published 6 articles were classified as duplicate. After performing our selection process, 17 studies were obtained from 1992 to 2017 (7-10, 13-23, 25, 26) (table 1). In 8 out of 17 (47%) articles, the researcher sent the patients a reminder (7, 8, 10, 13, 14, 17, 23, 25), in five (29%) articles, a reminder was sent to a physical therapist (9, 19-21, 23). In two (12%) studies, the care-taker team was sent a reminder (15, 16), while in one study, the family doctors were the target of the reminders (18), and in another one, the pharmacists were the target of the reminders (26). Among the articles reviewed, 8 (47%) articles were randomized controlled trials (7-10, 19-21, 23) and the remaining 9 (53%) were descriptive (13-18, 22, 25, 26). 

**Reminder instrument: **We found various reminder instruments utilized across the selected literature such as text messages, phone calls, letters, face-to-face contacts, or a combination. In 4 of the 17 (24%) articles letters were used to remind the patient, the physician or the medical staff (9, 16-18). In 3 (18%) of them, a text message was used as the data gathering instrument (7, 8, 25). Definitely in one paper, in case there was no response to the text message, the researcher would have to make a phone call (7). In 2 (12%) other studies, phone calls were used as a reminder instrument (10, 19). In 3 (18%) others, emails were sent (21, 22, 26), and in 1 (6%) article, a notice was put on the wall in the room where the patient was (23). Mixed methods were used in 2 of the 17 (12%) articles where letters and phone calls were used together as a reminder(15, 20) and in 1 (6%) study letters, phone calls, and face-to-face meeting were used simultaneously (13). However, in only one article, the reminder instrument was not clear (14). 

**Outcomes: **In six of the 17 (35%) articles, the researcher reported that the use of reminder positively influenced the outcomes (8, 9, 13, 21, 22 and 25). Increased rate of response to reminder ranged from 9% to 13%. In these studies, the objective was the collection of the questionnaires' responses or data which estimated the outcomes through participation percentage as well as the data collection procedure (8, 9, 13, 21, and 22).

In 9 of the 15 (60%) articles, the effectiveness or ineffectiveness of reminders was not reported (10, 14-20, 23). In 4 articles, the response rate was reported without referring to effectiveness or ineffectiveness of reminders in the outcomes (14-17). In one of these studies, the response rate in receiving a response from nurses, occupational therapists and physiotherapists was 58% (15). No significant difference was observed between the response rates of these three groups. Finally, the purpose of two studies was only to gather the data (7, 26). Details of studies in chronological order from 1992 to 2017 are presented in table 1.

**Table 1:** Studies included in the review in chronological order

**First Author**	**Subject**	**Method**	**Reminder instruments**	**Response Rate**	**Outcome**
McAlindon (14*)	2102 patients	Descriptive (structured and semi- structured questionnaire with open-ended questions and interview)	No mention	Response rate was 80.6%	After one reminder, the response rate was similar between gender and across the age ranges, although there were more women in the older age groups. Respondents with knee pain had significantly more disability relating to upper as well as lower limb activities
Hasvold (17**)	2409 patients	Descriptive (cross-sectional)	Letter	Response rate was 80.5%	The return rate was slightly higher among the oldest people. Gender distribution was the same among the responders and non-responders.
Snels (16)	Caretaker team:500 (Physiotherapists:100 Occupational therapists:100 Rehabilitation physicians:100 Nursing-home physicians: 100 Neurologists:100)	Descriptive (structured and semi- structured questionnaire with open-ended questions and interview)	Letter	Response rate for care-taker team is 70.2%	Most of the responding occupational therapists were females and that the majority of physicians were males. Physiotherapists, response rate 83% Occupational therapists, response rate 75% Physicians, response rate 75%, Nursing-home physicians, response rate 60% Neurologists, response rate=58%
Pomeroy (15)	Caretaker team:996 (Nurses: 332 Occupational therapists: 332 Physiotherapists:332)	Descriptive (structured and semi- structured questionnaire with open-ended questions and interview)	Letter and Phone call	Response rate was 57.8%	Non-respondents were sent a reminder with a copy sent to the link clinician. If the questionnaire was not returned within a two weeks or more than a researcher telephones the link clinician with a gentle reminder. Pilot questionnaires, Response Rate: 86% The main questionnaires, Response Rate:57.8% Response rates did not differ significantly between nurses, OTs, PTs. Response bias is thought to be minimal.
Bekkering (19)	113 physiotherapists and 500 patients	RCT (Clustering Randomization)	Phone call	o Mention	Intervention group received an additional active strategy consisting of a multifaceted program including education, discussion, role playing, feedback, and reminders. The active strategy moderately improved adherence to the guidelines. The adherence to all criteria was 42% in the intervention group and 30% in the control group.
Bishop (9)	900 Physiotherapists	Descriptive (cross-sectional)	Letter	Response rate increased 8.9%, from 48.8% to 57.7%	Three weeks after distribution of the main questionnaire, postcard reminders were sent to all non-responders. Finally, 6 weeks after the second mailing, 20% (n=80) of the non-responders were sent a further questionnaire.
Bishop (18)	462 Family physician and their patients	RCT (2 intervention group and 1 control group)	Letter	No Mention	Intervention groups received reminders summarizing the recommended guidelines. Each family physician received a “guideline reminder letter” at each of three separate stages of the patient’s clinical course. No significant difference was between control group and intervention group.
Bekkering (20)	113 Physiotherapists 247 Patient in intervention group 253 patient in control group	RCT (Clustering Randomization)	Letter and Phone call	No Mention	Active strategy (consisting of 2 sessions with education, group discussion, role playing, feedback, and reminders) in intervention group did not improve patient outcomes. Reminder to physiotherapists and patients.
Smith (23)	65 Patients Intervention group:31 They reminded by a notice on the walls Control Group:34 They did not remind	RCT (Blind Single)	Notice on the walls	No Mention	Majority subjects female no significant difference in age or cognitive score between the two groups No difference in exercise score between groups. Statistically significant small positive correlation between exercise score and cognition. As a written exercise sheet (reminder) did not reinforce the learning sufficiently, these patients clearly required a higher level of supervision and repeated teaching to remember the exercises accurately.
Bell-Syer (13)	RCT1: 87 general practitioners and 187 patients RCT2: 39 general practitioners and 240 patients	Descriptive (comparing tow RCT studies)	Letter, Phone call and face-to-face meetings	RCT1: Exercise Trial Response Rate: 73% RCT2: Acupuncture Trial Response Rate: 73%	Project updates, project reminder letters, personal practice visits and telephone calls were the most successful strategies according to the findings from the GP survey. Project acknowledgement, discharge letters were also reported as useful only in the acupuncture trial. Trial information posters in surgery waiting rooms were not useful in the exercise trial.
Smith (22)	306 Physiotherapists	Descriptive (National survey study)	Email	First response rate:33.3% In first reminder response rate: 45.7% (Increased 12.4%) In second reminding response rate: 58.8% (Increased 13.0%)	A final limitation of this study was the low response rate. 59% . Whilst this figure may be regarded as respectable for a postal survey, it remains unclear whether the remaining 41% had different experiences to the respondents. In order to optimize response rates, previous studies have recommended providing incentives Response rate may also have been increased through using a web-based questionnaire design
Taylor (8)	679 patients Intervention group:342 Control Group:337	RCT (Single Blinded)	SMS	Nonattendance in intervention group: 11% Nonattendance in control group: 16% (Significantly)	SMS reminder to patients before their appointment was effective in reducing nonattendance in physical therapy outpatient departments There were more women than men. (61%) No differences in nonattendance for the factor of sex. Nonattendance at the next scheduled appointment for patients with SMS reminder was 11% compared with 16% for patients without reminder. 19 SMS reminders were needed to be sent to prevent 1 missed appointment suggested that this system leads to economic savings. The cancellation rate for patients receiving an SMS reminder (20%) was observed to be non-statistically higher than the other group (15%) The attendance rate was same in 2 groups (69%) Major effect of reminders was to prompt people to cancel unwanted appointments. Patient characteristics independently associated with a higher nonattendance rate were younger age and neck and trunk musculoskeletal or neuromuscular disorder.
Macedo (7)	133 Patients	RCT	SMS(for data gathering, not for reminding)	No Mention	73% of the participants that suggests, using mobile phone technology such as SMS is a potentially viable option for data collection in a clinical research study. In participants who owned a mobile phone, the response rate was high (75%) and was not influenced by age, sex, education level, or severity of the condition. Overall, the responses to the SMS did not decrease over time and were consistent during the 12 months of data collection. SMS supplemented with phone interviews, but not SMS alone, is a feasible option to collect data within a back pain clinical trial setting.
Tan (10)	100 Patients	RCT (3 intervention group and 1 control group) Group 1: an eight-session self-hypnosis training intervention (HYP-8) without audio recording for home practice; Group 2: HYP-8) with recordings; Group 3: A HYP-2 with recording and brief weekly reminder telephone calls; Group 4: an eight-session active control intervention.	Phone call	No Mention	The hypnosis groups combined reported significantly more pain intensity reduction than the control group. There was no difference among the three hypnosis conditions. Improvement in pain intensity, pain interference and sleep quality did not differ among the three hypnosis groups.
Bernhardsson (21)	448 Physiotherapists Intervention group:277 Control Group:171	Non-randomized trial	Email	168 PTs (60.6%) in the Intervention group and 88 PTs (51.5%) in the control group responded to the follow-up questionnaire.	There were no differences in respondent and workplace characteristics within the groups between baseline and follow up. higher proportion of PTs in the intervention group (59%) than in the control group reporter being aware of guidelines, knowing where to find guidelines, and having easy access to guidelines. A tailored, theory- and evidence-informed, multi-component intervention for the implementation of clinical practice guidelines had a modest, positive effect on awareness of, knowledge of, access to, and use of guidelines, among PTs in primary care in western Sweden. In general, attitudes to EBP and guidelines were not affected.

## Discussion

The results of our systematic review show that the reminder methods used in the evaluated studies consisted of SMS, phone call, letter, email and notices on the wall. In spite of the higher cost of letter and phone call compared to SMS and email, more than 50% of studies used these methods as a reminder. Considering that more than 90% of the population in many countries have mobile phones (24), we suggest that the mobile phone be used as a potential device or a support electronic health reminder systems. Definitely, in some articles, the reminders via SMS have been assessed and considered effective. In 2014, Compere examined the impact of SMS on reducing absenteeism in preoperative anesthesia clinic appointments, and stated that this approach reduced the absence of patients and was also a cost-effective way (27). 

Keeshin (2017), examining the impact of SMS on the immune response to HIV viral infection of human papilloma in HIV positive young patients, concluded that SMS could have a positive effect on the immunization of these patients (28). SMS is a low-cost reminder method for a patient or specialist (27). Although in some studies, comparing the effects of various reminders such as postal mail, email, text, postcards, auto dialer and phone calls, the researchers conclude that calling is the most effective way as a reminder system (29, 30). Certainly in some studies, postal mail and phone calls have had the greatest impact as reminders (30) but as mentioned earlier, phone calls are considered expensive (31, 32). Ultimately, reminding with different tools cannot be fully effective, and definitely face-to-face reminders and the direct relationship with the patient are the most effective reminder methods (33) which of course, are never feasible and will certainly not be cost-effective.

According to present findings, more than one-third of reminder instruments have resulted in better outcomes showing the importance of reminders in patient treatment process. Various studies have shown the positive effect of reminders on the results. For screening type 2 diabetes among women with gestational diabetes mellitus (29, 34) reminder was effective and tended to timely diagnosis and prevention. Besides, the use of reminders has been effective in vaccine reminder alerts and has led to getting more vaccines on time and thus helps prevent future risks (30-32, 35). In addition, Meddings in a literature review, showed that using a reminder is effective on reducing the use of urinary catheters in hospitalized patients and consequently, reducing urinary tract infections (36). In fact, following-up patient’s treatment is highly important, particularly in physical therapy where therapeutic strategies such as exercise programs involve several sessions of treatments. It is worthy to note that in those studies that have used a reminder, the aim was to remind the patients of the appointment and some guidelines of the physiotherapists or caretaker teams (9, 15, 16, 19, 21, and 22). 

In other medical areas, the use of a reminder as a way is very useful. Cancellation of appointments due to forgetfulness has always been a recurring problem, and the reminder has been able to significantly remedy this problem, which has increased the timely availability of patients in their turn for diagnostic and therapeutic measures (27, 33, 37-39). Furthermore, the use of reminders for specialists to follow evidence-based guidelines is somehow very common. In some studies, an overview of the adherence of specialists to guidelines suggests that reminder systems can be effective in monitoring and continuously improving the performance of the doctors (40, 41). 

Additionally, reminders were effective on patients to observe medical orders especially timely medication taking in different ages tending to better outcomes (42-46). Although the use of very inexpensive means to remind drug use, pill bottle strip with toggles, digital timer cap and standard pillbox was not effective in an appropriate and timely drug administration (47). However, in only one study, a text message was sent to subjects aiming to determine the mean of monthly self-perceived pain in a year (7).

Importantly, in physical therapy, it is sometimes needed to remind the patients to do some exercises regularly or to regard some positions to gain better results. In fact, long term adherence to regular exercise is a difficult goal to achieve. The objective of one study was to send SMS to patients to increase self-management by doing some usual care physical therapy movements (25). Moreover, promoting patient empowerment for self-management in some other diseases including asthma, as well as the use of contraceptive drugs has been proven via reminders (48-50).

The study showed positive results for the patients. According to Jenkins, sending targeted reminders could be useful and tend to decrease diagnosis and treatment costs for the patients and health systems (51). The use of reminders is not always easy and there are various barriers in using it, including the lack of human resources to create a reminder system, management and updating reminders as well as financial resources (52). Likewise to set up a reminder system in each field, the system should be tailored according to the target group and pay particular attention to reminder content, type, number and time of reminder (37, 29). Reminder may not be effective in all areas. According to a review by Cooper on the impact of a reminder system on improving the rate of influenza immunization in children with asthma, which was done face-to-face, as the most effective way of reminding, and also by e-mail, it was concluded that reminders had a relatively low and unimportant effect on improving immunization rate in children with asthma (53).

Technological development in communication opens new ways for assessment and treatment strategies. For example, it is possible to send video files of exercises or text messages containing recommendations to patients guiding them to do daily activities more correctly, via internet or mobile phone. Furthermore, there is a possibility to organize groups of patients for discussing various matters regarding the patients’ special conditions and issues. The current review showed that new solutions in communicating with patients and physical therapist have to be researched in the future for their potential contributions.

In conclusion, reminder methods used in physical therapy consist of SMS, phone call, letter, email and notice on the wall. The inclusion of reminder in physical therapy trials may result in better overall outcomes. 

## Further Research studies

Considering the development of technology and the fact that 90% of the general populations have mobile phones, reminder procedures should be adapted to current technology scenario. 

## Funding:

There was no source of funding for this study.

## Conflict of interest:

We have no conflicts of interest to disclose.
